# Supplementary material for: Middle Eastern Genetic Variation Improves Clinical Annotation of the Human Genome
Source: J Pers Med. 2022 Mar 9;12(3):423. doi: 10.3390/jpm12030423 (PMC8956070; doi:10.3390/jpm12030423)
Supplement: Supplementary file 1 [file jpm-12-00423-s001.zip › Supplementary Figure S1.pdf]

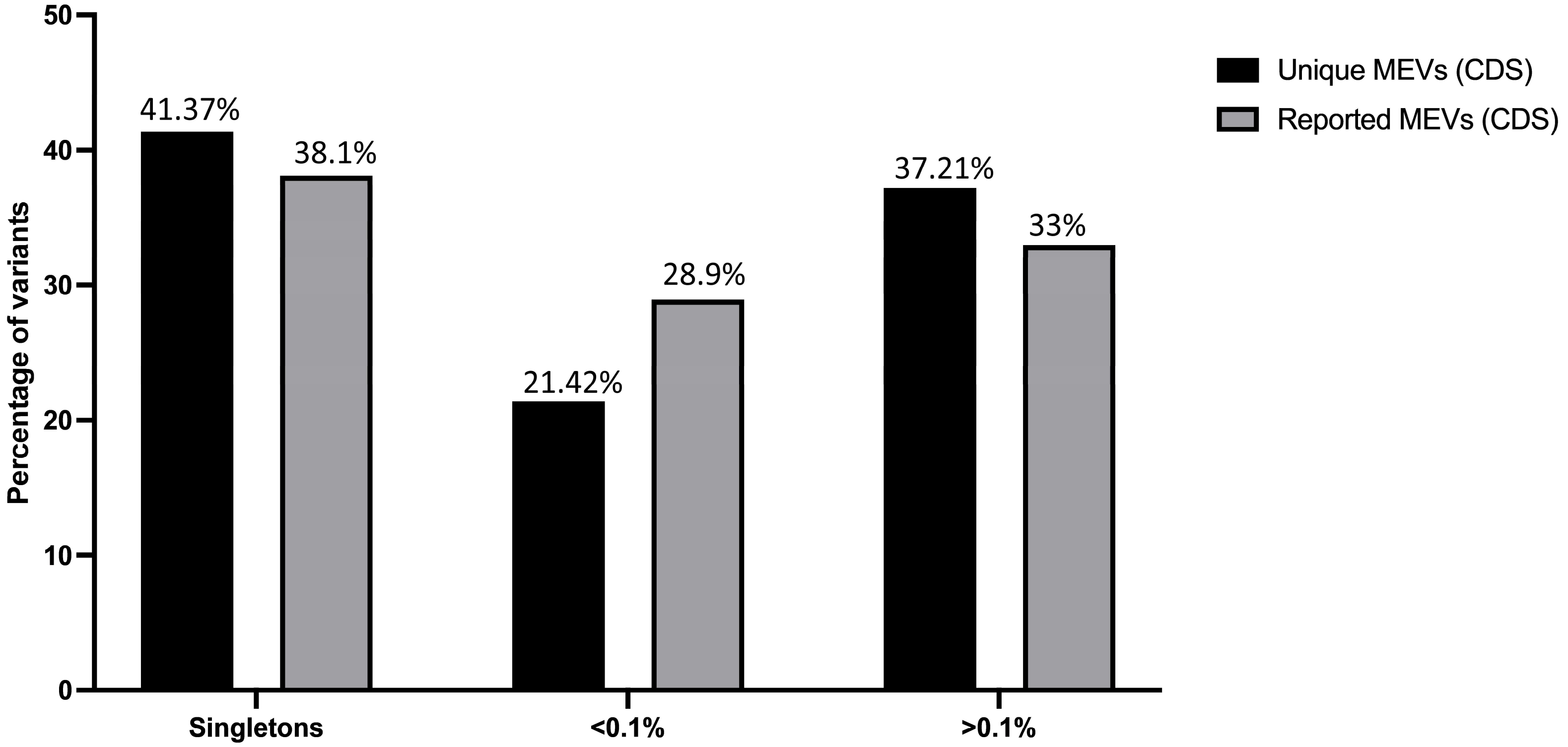


**Figure S1.** Distribution of Unique and Reported MEVs based on MAF. Compared to the reported coding variants, a larger proportion of the unique variants were singletons. Unique coding variants = Variants not reported in gnomAD 2.1.1 Exomes. Reported coding variants = Variants reported at least once gnomAD 2.1.1 Exomes. Singletons = single alleles observed in only one individual in the MEV dataset.
